# A Foreign Body as a Gynaecological and Sexological Issue—Case Study and Literature Review

**DOI:** 10.3390/medicina61020290

**Published:** 2025-02-07

**Authors:** Aleksander Frąckowiak, Stefan Sajdak, Grażyna Jarząbek-Bielecka, Klaudia Dolińska-Kaczmarek, Katarzyna Plagens-Rotman, Piotr Merks, Tomasz Kościński, Monika Englert-Golon

**Affiliations:** 1 Division of Gynaecological Oncology, Department of Gynaecology, Gynecological Obstetric Clinical Hospital, Poznan University of Medical Sciences, 33 Polna St, 60-535 Poznan, Poland; afrackowiak@ump.edu.pl (A.F.); mgolon@ump.edu.pl (M.E.-G.); 2 Poznan University of Medical Sciences, 10 Fredry St, 61-701 Poznan, Poland; ssajdak@ump.edu.pl (S.S.); kkaczmarek@ump.edu.pl (K.D.-K.); tkoscinski@ump.edu.pl (T.K.); 3 Collegium Medicum University of Zielona Góra, Zyty 28, 65-046 Zielona Góra, Poland; 4 Center for Pediatric, Adolescent Gynecology and Sexology, Division of Gynecology, Department of Gynecology, Poznan University of Medical Sciences, 61-758 Poznan, Poland; grajarz@o2.pl; 5 Department of Pharmacology and Clinical Pharmacology, Faculty of Medicine, Collegium Medicum, Cardinal Stefan Wyszyński University, 01-815 Warsaw, Poland; p.merks@uksw.edu.pl

**Keywords:** foreign body, vagina, menopause

## Abstract

*Introduction*: The most commonly encountered foreign body in the vagina is a tampon, which is routinely removed during gynaecological examinations. While these cases are common and well-known in medical practice, there are also situations involving much more unusual foreign bodies. This article focuses on these rare and often surprising cases, which can pose diagnostic and therapeutic challenges. Highlighting this topic aims to draw attention to the variety of situations gynaecologists may encounter in their daily professional practice. *Case Study*: A 62-year-old woman was admitted to the Gynaecological Obstetric Clinical Hospital of the Poznan University of Sciences to have a foreign body removed from her vagina. An MRI examination revealed a calcified well-defined structure (94 mm × 68 mm × 96 mm). The material removed during surgery were calcified fragments surrounding a plastic deodorant cap. After decalcification of the lesion, a histopathological examination ruled out cancerous cells. The patient received gynaecological and urological treatments due to a vesicovaginal fistula. *Conclusions*: The gynaecologists’ expertise and skills in diagnosing and removing foreign bodies are essential in order to provide postmenopausal women with comprehensive and responsible medical care.

## 1. Introduction

The issue of foreign bodies in different parts of the human body concerns not only gynaecology, but also paediatrics, paediatric laryngology, paediatric urology, and paediatric gynaecology. Children may insert game counters or toy elements into their nose, urethra, or vagina. Foreign bodies may also be inserted by adults, e.g., mentally disabled, those having mental or psychosexual disorders, or elderly people with dementia. Objects may be found in different parts of the body, such as in the genital area. It should be noted that foreign bodies may also be inserted by sexual abusers. In such situations, psychosexual diagnostics should be implemented in order to find out if the child or disabled person is not a victim of violence. Sexology has recorded cases of persons with sexual orientation disorders inserting foreign bodies for sexual satisfaction.

Foreign bodies are usually inserted by patients themselves at different ages, but the word ‘usually’ needs to be emphasised as they can be inserted by other people in an act of violence. As mentioned, the issue of foreign bodies also concerns gynaecology, and importantly, not only paediatric but also general gynaecology. Undoubtedly, among all gynaecological issues, vaginal foreign bodies are a relatively rare phenomenon in gynaecology. In the context of paediatric gynaecology, they are most common in young girls under the age of 4. They occur sporadically in reproductive and postmenopausal women and concern persons with the aforementioned comorbidities [1,2,3,4].

Foreign objects are significant clinical issues, which can lead to serious health conditions, e.g., vaginal discharge, chronic inflammation, abscesses, fistulas, urinary bladder or bowel perforation, and pelvic disorders. The first indicative symptom is often bleeding, but malignant tumours, mainly endometrial and cervical cancer, are taken into consideration in the first place [3,5,6].

Gynaecological literature has described cases of the presence of foreign objects such as tampons, hair slides, buttons, seeds, toy elements, objects used in foreplay, pessaries, ballpen caps, toilet paper, batteries, and illegal drugs. There have also been cases of foreign bodies, e.g., polyurethane foam, which remained in the vagina for a long time, resulting in serious complications and requiring a surgical intervention. The presence of calcified foreign bodies constitutes an additional challenge for physicians, particularly in the context of a diagnosis and removal [7,8].

In order to confirm the presence of a vaginal foreign body, an ultrasound examination is usually performed (nearly 80% sensitivity). In some cases, it is necessary to use Doppler techniques or radiography, but only a small percentage of cases required computer tomography, magnetic resonance imaging or vaginography. These were most often supplementary techniques applied before scheduled surgeries [9,10].

This report presents a case of a 62-year-old female patient who had a foreign body in the vagina for 12 years.

## 2. Case Study

In June 2019, a 62-year-old woman was referred to the Gynaecological Obstetric Clinical Hospital of the Poznan University of Sciences from another hospital where she had been treated for a femoral neck fracture. An X-ray examination revealed a calcified lesion in the vagina and further gynaecological examinations were performed.

Upon admission, inflammatory markers were within the norm—CRP and the level of leucocytes did not show any abnormalities. In laboratory tests, the level of parathormone was 16.7 pg/mL (reference range 15–57 pg/mL) and the level of calcium was at the upper limit of the normal range.

An MRI examination revealed a calcified well-defined structure (94 mm × 68 mm × 96 mm) of limited mobility, filling the entire vagina. A bimanual examination confirmed abnormal vaginal structure. The patient reported that 12 years ago her partner had inserted a deodorant into her vagina, but she did not remember what kind. Without any previous support from her partner, she felt embarrassed and did not ask for help in order to remove the foreign body, which remained in her vagina for many years.

During hospitalisation, the patient’s mental state evaluation was within normal limits. The patient did not require psychological consultation, nor were there indications for psychiatric treatment.

### Surgical Procedure

Due to the fact that it was impossible to remove the foreign body during a standard gynaecological examination, after having obtained the patient’s written consent, a surgery under general anaesthesia was performed. Considering the size of the lesion and its calcified character, an incision of the perineum at 7 o’clock was necessary to widen access to the tumour. The surgery consisted of breaking the calcified tumour down gradually and washing away its fragments from the vagina by means of physiological saline solution.

**Figure 1:**
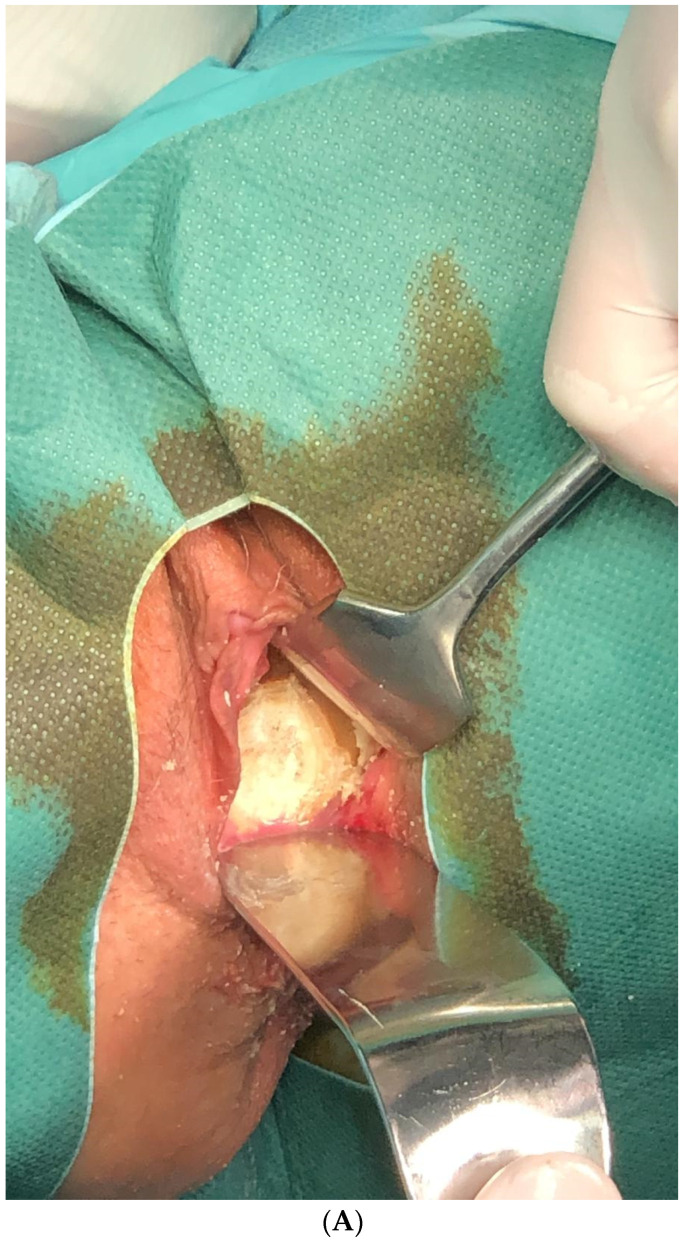

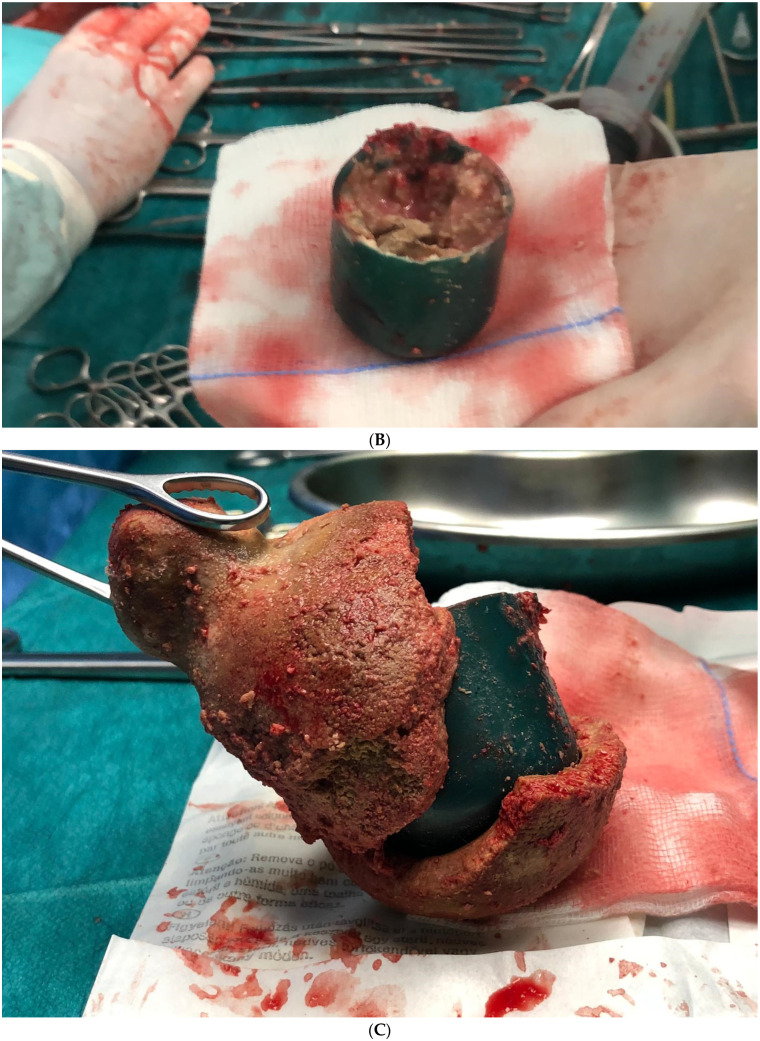

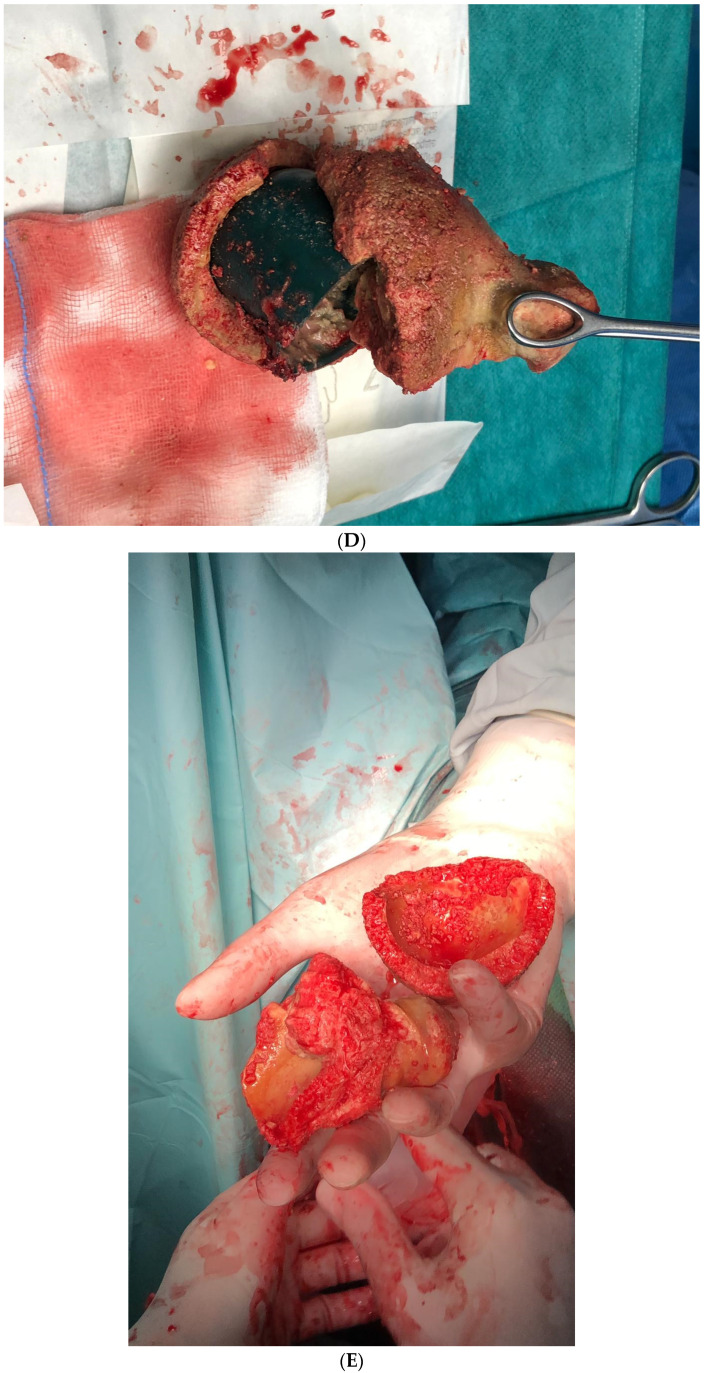

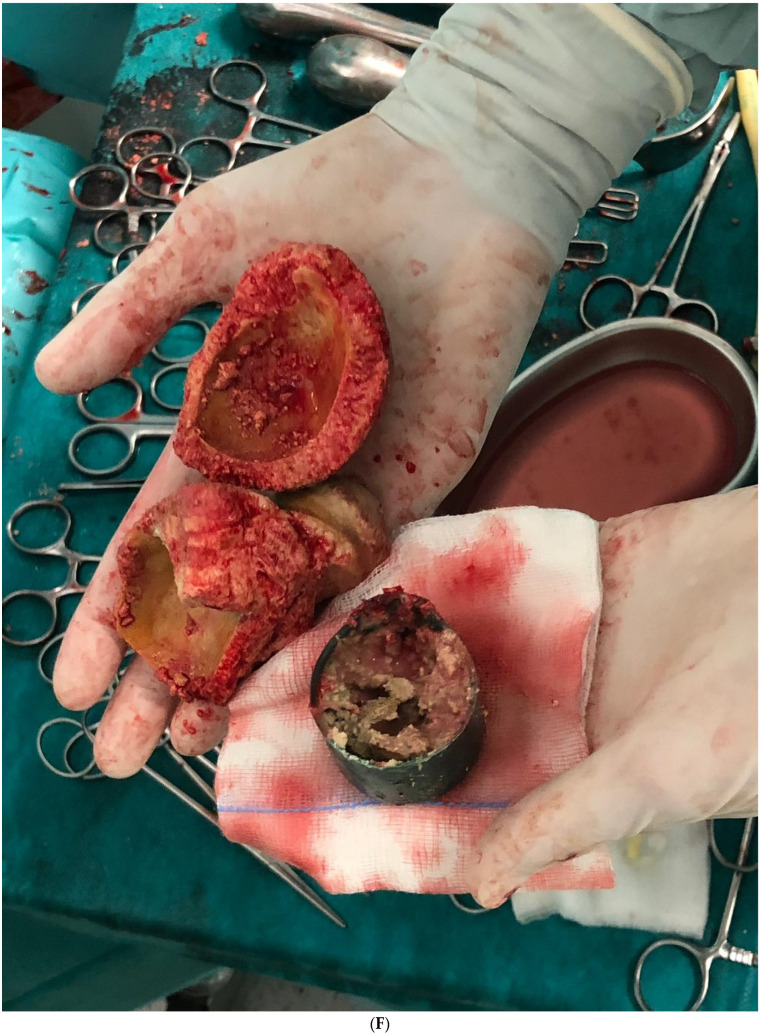

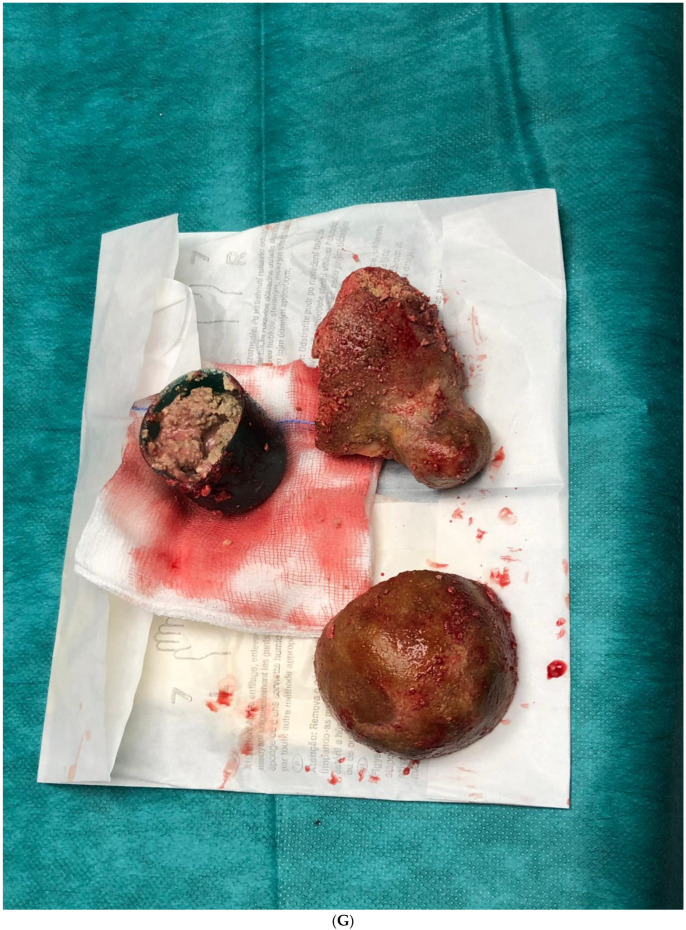
(**A**–**G**) Calcified fragments surrounding a plastic deodorant cap, removed from the vagina of a 62-year-old female patient (own material).

The removed material were calcified elements surrounding the plastic cap of the deodorant (Figure 1A–G). After decalcification of the lesion, a histopathological examination ruled out cancerous cells. The surgery was performed without any complications, but during the procedure a vesicovaginal fistula was found. After receiving postoperative care in the Gynaecological Obstetric Clinical Hospital of the Poznan University of Sciences, the patient was referred to the Department of Urology for further treatment.

In the Urology Clinic, the presence of a vesicovaginal fistula measuring 1.5 cm was confirmed. Due to the previous procedure for foreign body removal, persistent tissue swelling, and vaginal mucosal atrophy, it was decided to postpone the fistula closure surgery. A Foley catheter was placed and prophylactic antibiotic therapy and local estrogen therapy were prescribed. The patient attended regular urological follow-ups. After four months, complete spontaneous closure of the vesicovaginal fistula was observed, and the patient was informed that the previously planned surgery was no longer necessary.

## 3. Discussion

It turns out that even adult women with a foreign body are not aware that the object needs to be removed as it poses a threat to their health. They sometimes do not realise that they have a foreign body, e.g., when it is inserted into the vagina under the influence of alcohol or another substance, and this also concerns people with intellectual or mental disorders described above.

Having a vaginal foreign body is a specific and often embarrassing issue, being in the centre of interest of specialists in gynaecology and sexology. The prolonged presence of such an object can lead to serious health consequences, and in extreme cases to death. Among examples of vaginal foreign object are tampons or masturbation aids, and also cases related to violence, sexual abuse and different sexual preferences [11,12,13]. This issue concerns mainly children and elderly people with dementia who may be unaware of the consequences of their behaviour. There have also been cases of foreign objects inserted into the vagina by psychiatric female patients. Our patient, however, had not been diagnosed with any mental disorders or disturbances of consciousness.

Mędrala et al. [14] described a case in which diagnostics extended to imaging tests and a gynaecological examination revealed a connective tissue-covered cap of a popular multivitamin supplement in the vagina of a psychiatric female patient. In the course of an in-depth medical interview the patient admitted that 6 months earlier, during masturbation, a piece of a foreign body had become stuck in her vagina, but due to embarrassment and a subjective lack of alarming symptoms, she had not informed her physician of that fact. At the time of the application of the foreign body into her vagina, our patient was 50 years old. Puppo et al. [2], Chopra et al. [3], Ciebiera et al. [1] and Sharma et al. [15] have reported cases of women aged 74, 50, 70, and 73, respectively. All of them had foreign bodies retained for a long duration.

It should be emphasised that the majority of cases described in the literature concerned young women (median age: 22; range: 8–86). Scientific data available in the literature shows that children also constitute a large group. Nayak et al. [16] describe the diagnostic and therapeutic process in a 7-year-old girl with vaginal bleeding. An X-ray examination confirmed the presence of a sharpener inserted into her vagina one year earlier. In the case of vaginal bleeding, immediate diagnostics is indicated, particularly in order to rule out a malignant tumour or sexual abuse. It turns out that when there are differentiating causes of vaginal bleeding, one should take into account not only cervical pathologies and endometrium, e.g., endometrial cancer, but also the presence of a foreign body [16,17,18,19].

In our case, the foreign body was inserted into the patient’s vagina by her partner. A frequent form of violence against women is sexual coercion on the part of their husbands or partners, whose uncontrolled consequences can in extreme cases lead to death. In the case presented by Rancati, the use of genetic and forensic techniques helped in detecting elements of wood in the woman’s vagina, which turned out to be her husband’s limb prosthesis used as a tool of sexual coercion. The specificity of this case is an extremely non-standard object inserted in the victim’s vagina, i.e., a traditional exoskeletal lower limb prosthesis. The improper use of this object caused a large irregular lesion in the vagina, which was a direct cause of the woman’s death due to acute haemorrhagic shock [20].

An immediate removal of foreign objects helps avoid potential complications, e.g., vaginitis, urinary tract infection, vaginal wall ulceration, perforation, and vesicovaginal and rectovaginal fistulas [10]. Vesicovaginal fistulas caused by foreign bodies are extremely rare. Their occurrence in developing countries is related mainly to difficult delivery, iatrogenic injury during Caesarean section, and hysterectomy. A prolonged presence of a foreign body, which was the case in our patient, can lead to extreme complications. She reported this fact 12 years later and the object that was embedded in the vaginal tissue, migrated to the urinary bladder, caused considerable calcification and led to a vesicovaginal fistula. This made the removal and immediate correction of the injury more difficult. A continuation of urological treatment was required.

Mengistu [21] also described a case of a female patient who had a bottle cap left in the vagina for 2 years, which caused urine leakage and revealed a fistula between the vagina and the urinary bladder. A complex vaginal injury in a 7-year-old black Ugandan female was caused by a cassava stick (22 cm in length and 2 cm in diameter) left in her vagina for 6 months. It manifested itself in smelly vaginal discharge, and led to perforation to the small pelvis through the pouch of Douglas [22]. Nkwabong [23] described a very interesting case of a female patient having a nascent uterine myoma, which led to a vesicovaginal fistula manifesting itself by urine leakage that appeared 5 days following the myoma removal. Mengistu et al. [21] presented a case in which a fistula caused by a vaginal foreign object closed spontaneously after a two-week urinary catheterisation. A large number of situations require very complicated procedures and long convalescence.

Adult women are usually aware that a foreign object needs to be removed as it poses a threat to their health. A delayed medical intervention is most often caused by embarrassment. In the case of our patient, her husband was responsible for delaying the removal of the foreign body.

Among children and elderly people with dementia, a considerable delay in diagnosis is usually observed. A foreign body is often revealed by accident in the course of looking for causes of reported issues [16]. The issue of vaginal foreign bodies is also present in people under the influence of alcohol or abusive substances who have everyday objects, e.g., cosmetic caps, inserted into the body. After so many years, it is difficult to unequivocally state if this was a cause in our patient, but the case of the patient’s death after sexual abuse with her husband’s limb prosthesis concerned an alcoholic [20].

Contraception can be one reason for inserting a foreign object into the vagina. One method of treatment of a pelvic organ prolapse is pessary therapy. Literature data also describe cases of cervical injuries and fistulas developed due to an intrauterine device migration [9,17,24]. Sharma et al. [15] described the case of a 70-year-old woman complaining about recurrent uterine tract infections, smelly vaginal discharge, and sporadic abdominal pain. A gynaecological examination revealed a foreign body (ca. 10 × 10 cm) in the vaginal canal caused by pelvic organ prolapse treatment. Currently, plastic balls or some fruit used instead of an intrauterine device as non-pharmacological treatment of pelvic organ prolapse are unheard of. Imaging diagnostic methods constitute a valuable complement in detecting accurate spatial localisation of foreign bodies and the accompanying local inflammatory states [9,25]. In the case of our patient, the X-ray examination performed during differential diagnostics of the femoral neck fracture accidentally revealed the presence of the foreign body. It should be noted that a transvaginal ultrasound examination is considered the first line method in detecting vaginal foreign objects. In their retrospective cohort study among 249 female patients, Yang et al. [9] showed that this technique is characterised by 81% sensitivity and 53% specificity, with 100% detection ability when the diameter of a foreign body exceeds 5 mm. These data correlate with the analyses presented above. A valuable method that complements classic ultrasonography uses Doppler techniques, which show increased vascularity of the part of the vagina where a foreign body is located. It turns out that this method is useful in newborn diagnostics. As far as adults are concerned, it most often helps in differentiating benign and malignant lesions where the presence of a foreign body for many years may suggest malignant tumours at the first stage of diagnostics [9,10].

In the majority of situations, there is an unlimited availability of this method due to its common use in differential diagnostics of a variety of pathologies, e.g., those concerning endometrium and ovaries [26].

## 4. Summary

A vaginal foreign body may lead to dangerous inflammatory states and complications. It is worth noting that pessaries used by elderly women with dementia or an uncontrolled use of tampons during menstrual periods pose such a threat. Nevertheless, vaginal foreign bodies are also a gynaecological issue—they occur during “sex plays”, but also as a result of sexual abuse, which needs to be taken into consideration in gynaecological and sexological diagnostics. The issue of foreign bodies in the vagina often concerns young girls who usually insert them by themselves, but paedophile sex offenders should also be emphasised who prefer to insert foreign bodies in an act of violence.

## 5. Conclusions

Gynaecologists’ and obstetricians’ awareness of rare cases of the vaginal foreign bodies, particularly among postmenopausal women, is essential. Gynaecologists’ expertise and skills in diagnosing and removing foreign bodies are necessary in order to provide postmenopausal women with comprehensive and appropriate medical care.

Vaginal foreign bodies need to be reported and removed immediately as their prolonged presence in the vagina may cause serious complications.

## Data Availability

The original contributions presented in the study are included in the article, further inquiries can be directed to the corresponding authors.
